# Educational and personal impacts of the COVID-19 pandemic on emergency medicine resident physicians: a qualitative study

**DOI:** 10.1186/s12909-024-05972-4

**Published:** 2024-09-27

**Authors:** Elyse Fults, Jeffrey N. Gerwin, Michael W. Boyce, Melissa Joseph, Ambrose H. Wong, Leigh V. Evans

**Affiliations:** Department of Emergency Medicine, Yale School of Medicine, New Haven, Connecticut, USA

**Keywords:** Resident physician, Psychological stress, Emotiional experiences, COVID pandemic

## Abstract

**Background:**

The COVID-19 pandemic had a significant impact on both the clinical practice and the psychological states of frontline physicians in the emergency department. Trainees, at the beginning of their careers and thus still developing their practice styles and identities as physicians, were uniquely affected.

**Objective:**

In this qualitative study, we sought to explore how the pandemic environment shaped the experiences of emergency medicine resident physicians.

**Methods:**

This was a qualitative study. We conducted in-depth interviews with emergency medicine faculty, resident physicians, and staff at a single emergency department based at an urban academic institution in the northeastern United States. Interviews were audio recorded and transcribed, and transcripts were then analyzed in an iterative process by our coding team for recurring themes related to the resident experience.

**Results:**

We reached data saturation with 27 individuals. Of those who were interviewed, 10 were resident physicians [6 senior residents (PGY-3 or PGY-4) and 4 junior residents (PGY-1 or PGY-2)]. Three major recurring themes regarding resident physician experience emerged during our analysis of the interviews: (1) novel educational experiences dampened by negative structural forces from the pandemic, (2) fracturing of social interactions and mitigation through ad-hoc support systems and community of practice, and (3) development of negative emotions and psychological trauma including fear, resentment, and moral injury causing lasting harm.

**Conclusions:**

Our results suggest that emergency medicine resident physicians training during the COVID-19 pandemic faced unique experiences concerning their education, social support systems, and emotional states. While the educational and social experiences were described as having both negative and positive impacts, the emotional experiences were largely negative. Residency program leadership may use these insights to improve resident preparation, wellness, and resilience in the face of future adverse events.

**Supplementary Information:**

The online version contains supplementary material available at 10.1186/s12909-024-05972-4.

## Introduction

The COVID-19 pandemic influenced both the clinical practice and psychological states of frontline physicians in the emergency department (ED). With the spread of COVID-19 came increased patient acuity and a scarcity of important resources for patient care and healthcare worker protection. Several surveys of emergency physicians during the pandemic revealed increased levels of job dissatisfaction and burnout, decreased self-care, and even the development of post-traumatic stress disorder [1–3].

Although all emergency department providers faced the same pandemic environment, it stands to reason that trainees in emergency medicine, at the beginning of their careers and thus still developing their practice styles and professional identities, were uniquely affected. A growing body of research suggests that COVID-19 significantly impacted emergency medicine resident education and well-being (both physical and emotional). Waseem et al. surveyed 22 emergency medicine residencies in New York state and found that the majority of programs canceled off-service rotations and adopted virtual didactics, while half of programs stopped prospective research [4]. Another large survey by Weygandt et al. revealed a negative emotional impact on trainees including increased anxiety and difficulty with concentration in the educational environment [5]. Interestingly, studies have also identified positive effects of the pandemic on trainees. After conducting a mixed-methods survey at 7 emergency medicine residency programs, Ford et al. found that while trainees reported negative feelings about their education overall during COVID-19, they nonetheless experienced increased satisfaction with emergency medicine as a career choice [6].

Despite the highly valuable insights from existing research, studies evaluating the resident experience in a deep, exploratory qualitative approach during the pandemic have been limited [7, 8]. Qualitative inquiry into this topic offers an opportunity to delve deeper into a crucial period in medical education. In this study, we seek to further explore how the novel and volatile pandemic environment shaped the education and emotions of emergency medicine resident physicians. We specifically aimed to identify challenges and novel discoveries encountered by residents through their own narratives as well as the contextual narratives of others in the emergency department. By incorporating a breadth of experiences from both residents themselves and other front-line workers as a whole, our inductive analytic process provides a rich context of the resident experience. We hope to provide stakeholders with data-driven recommendations to improve resident preparation, wellness, and resilience in the face of future public health crises.

## Methods

### Study design

This was a qualitative study aiming to identify the educational and personal impacts of the COVID-19 pandemic on the training and experiences of emergency medicine resident physicians. It was part of a larger investigation seeking to determine factors affecting system responsiveness and clinician stress during the COVID-19 pandemic [9]. We conducted in-depth (30–45-minute) interviews with emergency department clinical staff with experience treating COVID-19 (and suspected COVID-19) patients. The research team consisted of an interprofessional group with expertise in psychology, human factors, systems engineering, and emergency medicine to provide a spectrum of perspectives and evaluate residents’ experiences in the emergency department setting. Our study protocol was approved by the Yale University Institutional Review Board. We followed the 32-item Consolidated Criteria for Reporting Qualitative Research [10].

### Participants and setting

Eligible participants included all front-line emergency medicine at a single emergency department of an urban, tertiary care, academic referral hospital belonging to a larger regional healthcare network in the northeastern United States. Front-line emergency medicine workers included resident physicians (PGY 1–4), attending physicians, nurses, and patient care technicians. Inclusion criteria for recruitment were front-line workers in roles listed as above who have worked at least one clinical shift caring for COVID-19 patients during the COVID-19 pandemic (March, 2020-November, 2021). We interviewed a diverse range of clinical staff roles rather than physician trainees alone in order to investigate the emergency department pandemic environment as a whole and contextualize the resident experience during this unprecedented time. Recruitment was conducted via departmental emails, announcements at regularly scheduled faculty and resident meetings, and in-person recruitment during clinical shifts. All participants provided verbal and written statements of informed consent at the time of recruitment and scheduling of the interview. Gift cards in the amount of $25 were given in compensation for completing the interview.

### Study protocol and data collection

As part of a larger study, interview questions were developed by our human factors expert and based on Normalization Process Theory (NPT) [11]. Interview topics were organized around the four core constructs within NPT (coherence, relations, operations, and appraisal) to explore barriers and facilitators of guideline adoption as well as factors contributing to clinician stress and burnout. In addition to the above questions, we included open-ended questions to explore the overall experiences and challenges of working during the COVID-19 pandemic. We piloted the interview guide on three volunteer participants with minor modifications made based on their feedback before the initiation of data collection. Please refer to Supplement 1 for a copy of our interview guide. Interviews were conducted in a semi-structured format allowing participants to freely express their views.

A member of our research team with expertise in user-centered design and process improvement research (J.M.R.) led the in-person interviews, and a second team member who was trained in qualitative data collection (J.G.) made field notes during the sessions. The interviewer shared an affiliation with the same department as the participants but did not have direct professional interactions with them in the clinical or academic domain. Interviews lasted approximately 30–45 min. All interviews were audio recorded and professionally transcribed, and transcripts were analyzed for themes by a six-member coding team (E.F., J.G., M.J., A.H.W., L.V.E, & J.M.R.) that included an emergency medicine resident, emergency medicine attendings, and research staff. A collaborative qualitative software package for thematic analysis (Dedoose, SocioCultural Research Consultants) was utilized for data organization [12].

### Statistical analysis

Transcripts of each interview were analyzed by the entire coding team for emergent themes pertaining to emergency medicine resident experiences during COVID-19. In order to create the code book, the coding team utilized a systematic, inductive approach. After an initial round of blinded open coding, additional codes were identified and integrated with existing codes through group discussions. As more information was added with additional interviews, a consensus was achieved on major themes through an iterative analytic process [13]. Transcripts were analyzed from all interviewees, and a primary codebook was developed. Codes were grouped and consolidated into major themes and subthemes as outlined and defined in Table 1. For this manuscript, we chose the most relevant and illustrative quotes specifically highlighting the resident experience.

**Table 1:** Key quotes illustrating themes

Theme	Subtheme	Description	Example
Novel educational experiences dampened by negative structural forces from the pandemic	Decreased patient volume and variety	Decrease in overall patient volume and “bread and butter” presentations was detrimental to resident clinical experience	“I felt like I wasn’t learning, ‘cause all I was seeing was COVID […] I felt those gaps in my knowledge for the end of intern year, I was kind of nervous going into second year” **(P26)**
	Decreased or restricted procedural experience	Procedural opportunities were decreased overall, and necessary procedures were often restricted to senior residents; subsequently there is concern that lack of procedural experience for juniors has caused a knowledge gap	“Unfortunately, I think some of our junior residents weren’t exposed to as many procedures earlier in training, and there might be a little bit of a knowledge gap now” **(P5)**
	Unique learning opportunities due to a novel disease	Training during a pandemic provided unique experiences and perspectives	“I think- at least I’m hopeful- that in 10 or 20 years, looking back on this- I graduated medical school during this global pandemic, and was put in this humbling, unique experience to work on the front lines. I think that’s something that will be a positive impact on me forever” **(P11)**
Fracturing of social interactions and mitigation through ad-hoc support systems and community of practice	Impact of isolation protocols	Isolation was especially detrimental for residents given the importance of bonding and support during training	“I’d go into work, and then, immediately, I come back home, decontaminate myself, and just stay at home because, at that point, absolutely no one was hanging out in person” **(P8)**
	Shared experience and strengthening of teamwork	The shared experience of navigating the pandemic brought residents closer together and helped alleviate some of the stress/trauma	“I feel like teamwork has been something in the emergency department as long as I’ve known it, before the pandemic. I think that’s one of the things that drew me to the specialty in general. I think the pandemic only exacerbated that and enforced that mentality. Just at the beginning, when people felt hopeless and unsure exactly how to manage the chaos, I think that was something that people could hold on to” **(P11)**
Development of negative emotions and psychological trauma causing lasting harm	Clinical uncertainty and unpreparedness	The newness and unpredictability of COVID-19 patients made residents feel stress and doubt about their skills	“I think the thing that sticks most to my mind is the extreme degree of uncertainty. You know? We didn’t know exactly what to do about these patients, what to do for them, didn’t know how to care for them […] there was a degree of uncertainty and nervousness” **(P19)**
	Fear for personal safety and transmission to others	Residents fear both their own safety and bringing illness home to their loved ones	“Is this a really serious, contagious infectious disease, that I’m at risk for contracting? What would that mean for me, not only personally in my health, but also as a carrier, am I possibly gonna put friends, families, or other patients at risk?” **(P5)**
	Resentment and lack of agency	Residents feel resentment that they are often the first/only providers to be exposed, but also feel that they do not have the agency to refuse	“[O]ther attendings required what I felt to be excessive degrees of patient contact. For example, diagnostic evaluations that only I could do. I actually became quite resentful that I felt that the job could be done one way, and then I would be exposed to more risk doing it the way that they wanted with I felt marginal patient benefit” **(P4)**
	Moral injury in patient care	Residents feel distressed due to practices leading to decreased patient interaction, physical touch, and visitor support	“There’s a lot of moral ambiguity, for lack of better words, in terms of treating COVID patients. How do you best advocate for the patient? How do you best advocate for yourself? How do you best advocate for the emergency department? A lot of times, all of the above are in conflict” **(P13)**

## Results

### Characteristics of study participants

We achieved data saturation after interviews with 27 front-line workers. Of those interviewed, 19 (70.4%) were female, and 24 (88.9%) had worked in the emergency department throughout the COVID-19 pandemic. Those interviewed were predominantly experienced healthcare workers with more than 4 years of experience (19 [70.4%]), and the majority of the participants interviewed were physicians (18 [66.7%]). A total of 10 resident physician participants were included, consisting of 6 senior residents (PGY-3 or PGY-4) and 4 junior residents (PGY-1 or PGY-2). All interviews were conducted over the course of a nine-month period, from March to November 2021. The characteristics of our participants are detailed in Table 2. A total of 53 individuals were approached: 17 (32.1%) physicians did not respond to the study request, while 9 (17.0%) potential participants did not follow up after multiple attempts to schedule an interview.

**Table 2:** Participant characteristics

Demographics of front-line workers interviewed	
Number of Participants, n (%)	27
Sex, n (%)	
Female	19 (70.4)
Male	8 (29.6)
ED experience, years (%)	
More than four	19 (70.4)
Less than four	8 (29.6)
Worked in the ED during the pandemic, n (%)	
Throughout	24 (88.9)
Part-time	3 (11.1)
Profession, n (%)	
Physicians (MD)	18 (66.7)
Resident	10 (55.6)
Attending	8 (44.4)
Nurse (RN)	7 (25.9)
Technicians & medical assistants (PCTs)	2 (7.4)

### Qualitative results

Qualitative analysis of these interviews identified three primary themes characterizing the experiences of emergency medicine residents during COVID-19: (1) novel educational experiences dampened by negative structural forces from the pandemic, (2) fracturing of social interactions and mitigation through ad-hoc support systems and community of practice, and (3) development of negative emotions and psychological trauma causing lasting harm. A summary of these themes, as well as their subthemes, definitions, and sample illustrative quotes, are provided in Table 1.

### Theme 1: Novel educational experiences dampened by negative structural forces from the pandemic

The healthcare environment during the pandemic, including the decrease in patients presenting for care and the increased precautions when caring for those patients, resulted in significant changes in educational opportunities for emergency medicine residents. Both clinical and procedural training were impacted.

Several respondents commented that the decrease in patient volume and variety was detrimental to their training experience. Because COVID-19 patients often predominated the presentations, the “bread and butter” chief complaints of emergency medicine, such as headaches and abdominal pain, were encountered less by residents. “I feel like there are some gaps in my knowledge of treating other things that I would have encountered earlier on,” one resident stated. “I feel like I shouldn’t have made it this far into my training without ever seeing croup or knowing what to do with croup, but here’s croup now.” (Participant 27) Another resident said, “Volume was down. Our very sick patients, like the heart attacks, stroke patients that we expect to see frequently […] the patients weren’t coming in.” **(P5)** Residents were also concerned that their clinical critical thinking skills were negatively impacted by the overwhelming amount of COVID-19 patients encountered. One reflected, “I feel like there’s big gaps in a lot of my critical care management […] if I couldn’t explain something, I said it was COVID. I perhaps falsely attributed symptoms for other diseases to that disease state.” **(P27)** Once the initial waves of the pandemic had passed and more patients began returning to the emergency department, the impact of changing patient volumes on resident training became evident. “The residents that started during the pandemic or immediately before the pandemic aren’t used to the volume,” one senior resident commented. “Because they are not used to the volume of the post-pandemic world, it’s been a bit of a learning curve trying to get to where it needs to be.” **(P13)**.

Because of isolation precautions, there was an effort to decrease the number of residents exposed to COVID-19 patients. This resulted in a relative scarcity of procedural training, especially with regard to intubation which has traditionally been considered an important area of expertise for emergency medicine physicians. “We limited the residents to senior residents only to do some of these higher-risk procedures, especially intubation,” one senior resident shared. “Some of the other residents actually weren’t able to do some of these procedures, and that may impact their clinical training.” **(P5)** Another resident echoed this fact, noting that “intubations, which were, I guess are often considered one of the highest exposure risk procedures, were restricted to certain classes of residents […] maybe three or four residents had done the vast majority of intubations.” **(P4)** As evidenced by these statements, junior residents were disproportionately affected compared to senior residents. “When [COVID-19] hit, the third and fourth years stayed in the ED, and they sent the juniors to the Peds ED,” said one resident. “They didn’t let them intubate. They didn’t want them—they were protecting them in many ways, but at the same time, they also didn’t have that steep learning curve like the seniors did.” **(P7)** This uneven distribution of experience caused stress for some individuals. “I think I’m probably gonna have a little bit of stress about feeling like I missed out on a lot of learning opportunities,” one junior resident admitted. “I think for a while I’m gonna feel like I am behind in terms of residency learning.” **(P11)**.

While many trainees felt negatively impacted by the educational circumstances caused by the pandemic, there were nonetheless some positive comments on the unique learning opportunities offered at such an unprecedented time. The severity of COVID-19 as a disease impressed upon residents the importance of considering and discussing patient goals of care, a crucial skill for physicians. “If you know how to talk about code status for a COVID patient, then you know how to do it for a cancer patient. Then you know how to do it for any other thing,” shared one resident. “Being able to do that quickly and everyone having that sense of duty, of fear aside […] that gives me hope that that’s what I trained for. That’s what they’re teaching us, and that’s what I’ll teach residents.” **(P7)** Being on the frontlines of a global pandemic inspired some residents to challenge themselves to become more capable and well-rounded. “Being able to assume more responsibility in my fourth year of residency was important,” said one senior resident. “I think it was an experience that COVID brought out, but I wouldn’t necessarily have had it if it wasn’t impacting our population. Being a little bit more aware of the resources and avenues for literature as they were being presented. Being a little bit more interested in reading cutting edge research and being able to interpret it in a way that we were able to actually translate it into changes in our clinical practice. Being adaptive.” **(P5)**.

### Theme 2: Fracturing of social interactions and mitigation through ad-hoc support systems and community of practice

Because of strict social distancing and isolation protocols during the COVID-19 pandemic, there was a significant decrease in opportunities for residents to spend time with others or on recreational activities outside of work. “I hated the isolation,” one resident said. “I think it was the worst thing […] I was going on three walks a day, and just to get fresh air, and have something to do on my days off.” **(P26)** Residency social events such as orientation activities and class dinners were not occurring as they had in the past, and many felt that this negatively impacted their ability to form relationships with peers. “I made it well into January and February before I saw some of my co-residents, which I feel like just created a very different bond than maybe other years had,” shared one junior resident. **(P27)** The failure to get to know one another as individuals also impacted individual residents’ identities and emotions in the workplace. “I envisioned in my mind being more social with the other people that I work with, getting to know each other better,” one resident said. “Maybe on some level I think I would have felt more confident trying to, like- or feel less need to prove myself because I feel like if you had accepted me socially, I could be a little more real with you. Whereas here I feel like I’m more of a unidimensional doctor resident character.” **(P27)**.

Several faculty shared concerns that this isolating environment would have long term negative effects on residents. “I am really worried about people in training right now […] who have just been profoundly isolated during really stressful circumstances,” one attending physician lamented. “I’m really worried about that, and I think that is a lasting stressor. I think that’s really colored people’s experience of training because everyone has been in survival mode for the last year. That’s fine, but there’s only so long you can do that.” **(P3)** This individual emphasized the importance of socializing to cope with the stress of working in the emergency department and noted that residents were lacking this opportunity. “Emergency medicine is stressful because people work long hours, and they see really terrible things happening to people,” they said. “I think for our emergency medicine trainees, especially, you have to be able to go out after a bad shift and talk about it and relax and sit with your friends, especially the people who came here as interns in the middle of a pandemic and missed out on those socializing opportunities, which I think are so critical for bonding and having someone to commiserate with. I think that’s gonna have lasting mental health impacts on people.” **(P3)** Some faculty reflected on their own actions (or inactions) with regard to resident interaction.

As with educational changes, however, there were also positive reflections on the social ramifications of COVID-19. Residents spoke of the increased support and teamwork that emerged from the shared experience of working during the pandemic. Although they were not able to spend time together socially, they did face the same challenges and stressors in the emergency department. One resident reflected, “I think our sense of unity and our allegiance to one another was one of the most important and remarkable components of especially the first couple of waves. I think we were all in the trenches together.” **(P4)** Another resident credited work relationships with perseverance. “[R]elationships with co-residents. Absolutely having other providers in that environment, dealing with the stresses […] they definitely helped deal with some of the psychological issues with it. Having a team that we can commiserate with, that you can have the shared, stressful emotional experience with definitely helped prevent any long-term trauma.” **(P5)** This sense of comradeship extended beyond just residents to other team members in the emergency department. “Something that I think worked well throughout this entire process was just the comradery between the physicians and residents and nurses, techs,” one resident said. “I think everyone was—had this all-hands-on-deck mentality. I think that that was something that really pulled us all through in terms of being able to best care for these patients.” **(P11)**.

### Theme 3: development of negative emotions and psychological trauma causing lasting harm

The emotional impact of working on the frontlines during the COVID-19 pandemic was one of the primary topics discussed by emergency medicine residents. Several negative emotional experiences were shared during interviews.

Many residents expressed doubt over their knowledge and skills when facing the unpredictability of COVID-19. Often this is related to the nature of COVID-19 as a novel pathology with no established clinical guidelines. “From the very beginning when patients started to show up and we were hearing about this and we were seeing things that made us concerned, I was the one caring for them, and people that were in my training level,” one resident shared. “It was the first time I saw somebody that was saturating at 60% but talking to me in full sentences and acting like that was not a big deal, which really throws your entire concept of medicine as you knew it up that point upside down.” **(P6)** Another said, “[T]he beginning was scary because you didn’t know anything, and everyone looks like they’re dying all the time […] You don’t know how to use your skills.” **(P7)** In some instances, however, there was a more personal and general reflection on readiness to become a physician. One resident admitted, “I thought there was gonna be so much more knowledge, I thought I would have so much more certainty […] when I imagined being a doctor.” **(P27)**.

Like other frontline workers, residents expressed strong feelings about exposure to a new and potentially dangerous disease. A frequent topic was the fear for personal safety and the safety of loved ones. “One of my biggest fears,” said one resident, “was not only me getting sick, but just bringing it home to my family. My wife never left the house. If she got sick, I knew there was only one person who brought it home to her, that was me.” **(P6)** Because of this sense of culpability, many went to extremes to protect their families at home. “I basically decon at the hospital, decon outside of my own apartment, sleeping in a different space than my partner. The whole thing was horrible,” recalled another. “It was ever-present. It was there at work and when I came home and I was worried about it all over again. In some ways more worried about it, because I’m bringing it home to an innocent person.” **(P4)**.

Residents also expressed resentment over being so exposed, yet they felt unable to refuse. “I think that there was a lot of hard feelings about being the ones in the rooms, being the ones exposed,” one resident shared. “There was a certain amount of, ‘I couldn’t not do this even if I wanted to not do this.’ [Y]ou don’t get to say no. They tell you to work, you work. It’s not like you can change residencies. It’s not like you’re gonna just not be a doctor, one year from finishing, because you feel unsafe.” **(P7)** This sense of obligation and responsibility among residents also limited their opportunity to process their emotions. “You just keep going. I remember thinking I don’t have time for my feelings about this. This is what I signed up for. I can’t change it. I can’t exactly call out. I’m doing the best I can with whatever I have […] that was probably more stressful than I realized it was.” **(P7)**.

At the same time, many residents felt distressed at the type of care being provided to their patients. Isolation protocols limited physical patient contact. “We treated these people- and we had to- like they were radioactive. You know what I mean? We didn’t wanna touch them,” one resident lamented. “It just really, I felt like dehumanized medicine for a long period of time.” **(P6)** In some instances, residents believed that appropriate patient care was sacrificed for the sake of these protocols. “I feel bad for the patient because I felt like we weren’t doing everything we could for him,” another resident shared. “[N]ot being able to offer a service because of the tragic circumstances really stuck with me […] Then a sense of failure that we failed this patient, we didn’t necessarily do everything we could for him, and being frustrated with the process.” **(P5)** The no-visitor policy also struck residents as disheartening. One resident described a common conversation with patients, saying, “I know that there were just so many instances that they were, like, ‘Sorry, we can’t have your family back.’ I’m just, like, ‘This is insane.’ Like, this is inhumane to not be able to bring someone’s loved ones back.” **(P26)**.

## Discussion

Our results demonstrate that emergency medicine resident physicians during the COVID-19 pandemic faced distinct experiences with regard to their education, social support systems, and emotional states. Although unique educational opportunities were presented as a result of the pandemic, overall resident training was negatively affected, and significant psychological stressors caused lasting emotional trauma. The experiences of residents are important to study because they are especially vulnerable to changes in the clinical environment and represent a critical component of the future healthcare workforce. Furthermore, because of their status as trainees in the traditional power structure of the healthcare environment, they often have the least agency to make their voices heard and advocate for change.

Unfortunately, the potential for positive educational changes due to the pandemic was overshadowed by the negative consequences of abrupt and severe changes in healthcare delivery due to public health measures. Some respondents found that the pandemic inspired unique educational growth, including improved patient communication, literature awareness, and adaptability to challenging situations. However, decreased patient volume and variety resulted in residents feeling unprepared to handle the “bread and butter” presentations of emergency medicine, such as heart attacks, strokes, and croup. Likewise, a decrease in procedural opportunities was believed to cause essential skills gaps. Retrospective studies conducted recently have confirmed a decrease in the number of procedures performed by residents across multiple emergency medicine residency programs [14, 15]. Our data suggests that junior residents were particularly affected by these changes, and other research has similarly noticed this discrepancy between training levels [6]. The impacts of these resident experiences during COVID-19 are relevant not only to residents themselves but also to the field of emergency medicine as a whole. From an educational perspective, the lack of clinical variety and procedural experience raises the question of whether residents, especially junior residents, have been sufficiently trained and confident to practice independently after residency.

Social isolation and disruptions in daily interpersonal bonding experiences severely impacted resident physicians’ well-being, but a silver lining developed to mitigate those negative effects due to the inherent team-based and collaborative culture innate to the field of emergency medicine. Many respondents lamented the lack of social interaction between peers and believed that this would have detrimental effects on mental health and future bonds between residents. Similar juxtapositions have been demonstrated in other studies [6, 7]. As with educational changes, junior residents seemed to be disproportionately detrimental by the inability to socialize and form support systems. The early parts of residency are crucial for forming bonds with co-residents and mentorship connections with senior residents and faculty, and these opportunities were limited. On the other hand, the increased sense of teamwork and mutual support that occurred organically among co-workers in the emergency department was appreciated. Emergency medicine is inherently a team-based specialty, and the stressors of navigating clinical and structural challenges during the pandemic together only enhanced that mentality.

Emotional impacts were described as largely negative. Residents shared feelings of doubt over their clinical abilities in the face of COVID-19. Senior residents, like more experienced emergency physicians, were struck by the uncertainty of COVID-19 as a disease process and felt ill-equipped to care for these patients. Junior residents’ insecurity was more general, having not yet been exposed to many of the typical clinical presentations seen in the emergency department. One of the most prevalent emotions among all residents interviewed was fear of either contracting the disease themselves or spreading it to loved ones, and this concern for personal safety has been a common theme in prior research [5–7]. Certain unique concepts were revealed in our interviews. Some residents felt resentment over being disproportionately exposed to COVID-19, while at the same time feeling that they had a frustrating lack of agency to refuse to work. Residents also spoke of the moral injury experienced in failing to provide the best care to their patients, whether because of isolation protocols or visitor policies. Prior research has discussed emotional reactions (specifically negative and reflexive emotions) as protective emotions used to assist in the resiliency and coping skills of the resident [8, 16]. From a social and emotional perspective, these various emotional experiences may shed light on increasing levels of burnout in residents as well as the lack of medical students applying to and matching into emergency medicine residencies in recent years.

In order to address these challenges and be prepared for similar events in the future, we believe there are actions emergency medicine residency programs can take. Simulation-based education is an ever-growing field and can help to supplement learning when there are experiential gaps in the clinical environment; it has been shown to improve mastery in healthcare trainees and provide practice for situations rarely encountered clinically [17, 18]. For example, many institutions implemented virtual or hybrid-based simulation training for resident emergency physicians to augment their clinical experience and round out their procedural and decision-making skills during and after the pandemic [19]. In order to maintain social support during future periods of isolation or unpredictability, structured peer and mentorship connections can be put into place. Some institutions, for example, had residents participate in a “Battle Buddy” system, wherein residents are paired up and responsible for checking in on their partner throughout the year [20]. Finally, we implore residency programs to prevent and address negative emotions that may arise in their trainees. This can be achieved in a number of ways, including regular debriefing of events, peer support groups, and providing resources for various options for counseling [21].

### Limitations

Our study does have several limitations. First, a number of clinicians did not respond to the study request or ultimately chose not to participate. There may be some self-selection bias, and it is possible that our participants had different experiences than those who chose not to participate in our study. Second, it is possible that the time between significant events and our interview could have affected the recollection of the experience. Memory processing and memory decay over time could affect the recall of the event as well as the emotions surrounding the event. Finally, it should be noted that these experiences are sampled from the experiences of clinicians from the same residency program at a single clinical site, and these experiences might not be wholly representative of resident experiences across the country.

## Conclusion

Our qualitative study demonstrated that emergency medicine resident physicians were profoundly impacted by their experience training during the COVID-19 pandemic. While educational changes and social circumstances were viewed both negatively and positively, the pandemic’s emotional impact was overwhelmingly negative. The ultimate long-term effects of the pandemic on physician practice and wellness have yet to be elucidated. However, the subsequent increase in emergency physicians leaving the profession and decreased number of applications to emergency medicine residency emphasizes the urgency of addressing similar challenges in the future. We hope that the themes uncovered here can contribute to the growing narrative on this topic and provide preventive solutions in future pandemics, large structural stressors on healthcare, or other natural or public health disasters.

## Electronic supplementary material

Below is the link to the electronic supplementary material.

Supplementary Material 1

## Data Availability

The data supporting the conclusions of this article is available upon reasonable request. Please contact Leigh V. Evans, MD at leigh.evans@yale.edu.

## Abbreviations

ED: Emergency Department
NPT: Normalization Process Theory
PGY: Post-Graduate Year
