# Supplementary material for: Educational and personal impacts of the COVID-19 pandemic on emergency medicine resident physicians: a qualitative study
Source: BMC Med Educ. 2024 Sep 27;24:1055. doi: 10.1186/s12909-024-05972-4 (PMC11429862; doi:10.1186/s12909-024-05972-4)
Supplement: Supplementary file 1 — Supplementary Material 1 [file 12909_2024_5972_MOESM1_ESM.docx]

**CRI:SIS Interview Guide**

**Welcome**

- Introduce self (interviewer) and note taker/observer
- Review informed consent
- Review process of this focus group/interview
- Review overall process of the study
- Explain value/richness of qualitative findings
- Thank participants for volunteering

**Process**

- Will last approximately 45 minutes
- Audio and video taped with Zoom with additional audio taping by note taker/observer: *introduce RA/observers
- Confidentiality
- Go to restroom if needed
- Turn cell phones off if possible
- Feel free to ask for clarifications
- Speak candidly and include details or events that illustrate your point as needed
- Want to hear both good and bad priorities/experiences
- Enjoy the experience
- Announce facilitator and participant

***Begin audio recording ***

- Any questions before starting?

**Interview Questions**

**What is your role in the clinical setting (attending/resident/RN/etc.)**

**Have you worked in the clinical care setting throughout the pandemic?**

- If no, when did you return to clinical care?

**Let’s start with your experiences caring for COVID+ or suspected positive patients in the ED. Can you tell me about an experience that stands out to you as memorable from early in the pandemic?**

- What challenges did you experience in caring for these patients at that time?

- What aspects of the care process worked well or facilitated better care at that time?

- probe for stress factors – what events or experiences either in the clinical care environment or outside of clinical care left you feeling stressed, either in the moment or reflecting back on the experience?

-probe for systems factors, clinical uncertainty, personal safety, etc.

**Can you tell me about a more recent experience caring for a COVID+/PUI patient?**

- What month/time frame was this experience?
- What stands out to you as different caring for these patients currently compared to early in the pandemic?
  - What aspects are easier or more difficult?
  - How has care for COVID+ patients changed now compared to earlier in the pandemic? How has that affected your stress?

Coherence: sense-making of new practices

**Tell me about your routine patient care these days…how have COVID and new guidelines implemented during COVID impacted your routine care practices? Can you give us an example or a recent experience that highlights that?**

Collective action: operationalizing a new practice, how will the work get done? Reflexive monitoring: ability to reflect on a new practice

**We recognize that the presentation of COVID-19 generated many changes in care processes and guidelines. Can you walk me through a recent or memorable experience with these changes. How has that changed over the course of the pandemic?**

- How have these guidelines impacted your practice at the bedside?
- What has been most challenging about the changes
- What has worked well with the changes
- How have you engaged in the process of guideline changes?

**Tell me about your most stressful experience with patient care during this time**

**How have these types of experiences impacted you personally…**

**Are there particular instances that come to mind that you carry with you?**

Cognitive participation: building working relationships around new practices, who will do the work?

**We know that Emergency Medicine relies heavily on working as a team. Tell me about your experiences with teamwork while treating COVID-19 patients.**

- How have the changing guidelines impacted teamwork or communication in your experience?
- How has the healthcare system helped or hindered you in your day to day experience as a clinician? Can you give an example?

**Do you have any recommendations for solutions or changes that can decrease stress for you and your colleagues in emergency medicine?**

**What things in the future may or may not cause you stress now as you think about the evolution of the COVID-19 pandemic?**
